# Effects of acute glucocorticoid blockade on metabolic dysfunction in patients with Type 2 diabetes with and without fatty liver

**DOI:** 10.1152/ajpgi.00030.2014

**Published:** 2014-08-07

**Authors:** D. P. Macfarlane, P. J. Raubenheimer, T. Preston, C. D. Gray, M. E. Bastin, I. Marshall, J. P. Iredale, R. Andrew, B. R. Walker

**Affiliations:** ^1^University/BHF Centre for Cardiovascular Science, Queen's Medical Research Institute, University of Edinburgh, Edinburgh, Scotland, United Kingdom;; ^2^Scottish Universities Environmental Research Centre, University of Glasgow, Glasgow, Scotland, United Kingdom;; ^3^SFC Brain Imaging Research Centre, University of Edinburgh, Edinburgh, Scotland, United Kingdom; and; ^4^University/MRC Centre for Inflammation Research, Queen's Medical Research Institute, University of Edinburgh, Edinburgh, Scotland, United Kingdom

**Keywords:** nonalcoholic fatty liver disease, Type 2 diabetes, glucocorticoids, fatty acid metabolism

## Abstract

To investigate the potential of therapies which reduce glucocorticoid action in patients with Type 2 diabetes we performed a randomized, double-blinded, placebo-controlled crossover study of acute glucocorticoid blockade, using the glucocorticoid receptor antagonist RU38486 (mifepristone) and cortisol biosynthesis inhibitor (metyrapone), in 14 men with Type 2 diabetes. Stable isotope dilution methodologies were used to measure the rates of appearance of glucose, glycerol, and free fatty acids (FFAs), including during a low-dose (10 mU·m ^−2^·min^−1^) hyperinsulinemic clamp, and subgroup analysis was conducted in patients with high or low liver fat content measured by magnetic resonance spectroscopy (*n* = 7/group). Glucocorticoid blockade lowered fasting glucose and insulin levels and improved insulin sensitivity of FFA and glycerol turnover and hepatic glucose production. Among this population with Type 2 diabetes high liver fat was associated with hyperinsulinemia, higher fasting glucose levels, peripheral and hepatic insulin resistance, and impaired suppression of FFA oxidation and FFA and glycerol turnover during hyperinsulinemia. Glucocorticoid blockade had similar effects in those with and without high liver fat. Longer term treatments targeting glucocorticoid action may be useful in Type 2 diabetes with and without fatty liver.

glucocorticoids, principally cortisol in humans and corticosterone in rodents, play a key role in regulating substrate availability and cellular energy balance (1, 25). However, although promoting lipolysis in adipose tissue and gluconeogenesis in liver may promote survival in times of acute stress or starvation, in the longer term, prolonged exposure to excess glucocorticoids may be maladaptive, with adverse metabolic consequences including the development of Type 2 diabetes (7, 41). Glucocorticoids promote hyperglycemia via a number of mechanisms: impairing insulin secretion (18), modulating glucose delivery to tissues (26), and inducing hepatic and peripheral insulin resistance (34). In addition, the metabolic consequences of glucocorticoids on fatty acid metabolism are likely to be highly dependent on prevailing insulin levels (8), such that relative insulin deficiency, as seen in Type 2 diabetes, may lead to more pronounced abnormalities of fatty acid metabolism, potentially exacerbating the contribution of glucocorticoids to peripheral insulin resistance (4).

Rodent studies suggest that modulation of glucocorticoid receptor activation may influence hepatic triglyceride accumulation (20, 23, 32) and the development of nonalcoholic fatty liver disease (NAFLD), a common feature in Type 2 diabetes (42). NAFLD is widely regarded as the hepatic manifestation of the metabolic syndrome (27) and is associated with hepatic insulin resistance (16), although it is unclear whether this represents a primary or secondary phenomenon (6). Metabolic tracer studies in nondiabetic individuals with NAFLD suggest insulin resistance in adipose tissue may drive the development of NAFLD, leading to increased release of free fatty acids (FFAs) and glycerol into the circulation, and promoting the accumulation of adipose-derived FFAs within the liver (5, 11). Glucocorticoids modulate these key pathways, but it is unknown whether abnormalities of glucocorticoid signaling exacerbate the development of NAFLD in Type 2 diabetes.

There is evidence that glucocorticoid receptor activation can be reduced selectively and safely within liver and adipose tissue by reducing local conversion of inactive cortisone to active cortisol by using an inhibitor of the enzyme 11β-hydroxysteroid dehydrogenase type 1 (11β-HSD1) (12, 35). However, the magnitude of effect on glycemic control after 3 mo of 11β-HSD1 inhibition in unselected patients with Type 2 diabetes was small. Recently, it has been shown that a selective 11β-HSD1 inhibitor lowers liver fat content by ∼2% after 3 mo administration to nondiabetic subjects with liver fat content >5.56% (37), raising the possibility that targeting antiglucocorticoid therapy at individuals with NAFLD may usefully stratify higher responders to 11β-HSD1 inhibitors.

To investigate the metabolic effects of reducing cortisol action in individuals with Type 2 diabetes we have tested the effects of acutely reducing cortisol action on the key metabolic pathways regulated by insulin. We used a “glucocorticoid blockade” approach as previously reported (40), by combining a GR antagonist (RU38486, mifepristone) with a cortisol biosynthesis inhibitor (metyrapone). Given the potential effect of glucocorticoids on pathways promoting NAFLD, we also quantified liver triglycerides by magnetic resonance spectroscopy (MRS), so that we could infer from a subgroup analysis whether patients with a high liver fat content are more sensitive to glucocorticoid blockade.

## MATERIALS AND METHODS

### 

#### Participants.

Fourteen patients with Type 2 diabetes were recruited from our hospital diabetes clinic. Given the abortifacient properties of mifepristone, and to avoid the influence of gender on glucocorticoid signaling, only male patients were studied. Inclusion criteria included: age 20–70 yr; HbA1c <8% (64 mmol/mol); BMI <40 kg/m^2^; negative tests for hepatitis B and C; general health good; normal screening blood tests (full blood count, renal function, electrolytes, thyroid function, ferritin); alcohol intake <20 units/wk; no medications known to increase liver fat, e.g., nucleoside analogs, methotrexate and amiodarone; no medications known to interfere with lipolysis, e.g., beta blockers; and no glucocorticoid use in the last 6 mo. All patients were receiving treatment with a single oral antidiabetic agent (metformin) and primary prevention with a statin. Importantly, neither metformin (29) nor statins (19) influence fatty acid turnover. Fat mass was measured by bioimpedance [body fat monitor BF302, OMRON Healthcare (UK), Henfield, UK]. Local ethical committee approval and written, informed consent were obtained.

#### Study design and protocol.

Participants entered a two-phase randomized double-blinded placebo-controlled crossover study of glucocorticoid blockade. In the active phase, subjects took 400 mg RU38486 (mifepristone, Exelgyn, Henley-on-Thames, UK) and 1 g of metyrapone (metopirone, Alliance Pharmaceuticals, Chippenham, UK) at 2300 the evening before and at 0800 on the morning of the study, as previously described (40). Study visits were separated by a washout period of at least 2 wk.

**Fig. 1.**
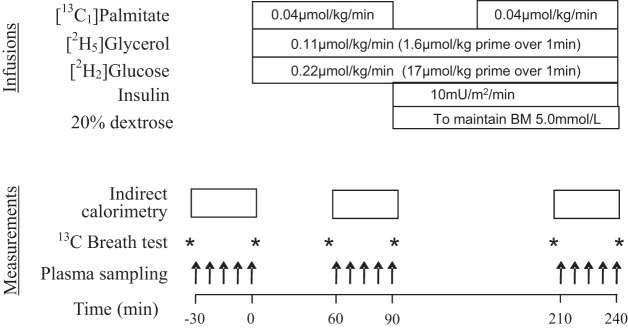
Study protocol. The insulin infusion was primed at 20 mU·m ^−2^·min^−1^ for 10 min. Asterisks mark time when breath sample was taken.

On each study day, subjects attended the clinical research facility at ∼0800 following an overnight fast. Subjects were asked to refrain from vigorous exercise and alcohol for 48 h prior to the study and maintain their normal diets. Studies were performed in a temperature-controlled room and subjects were studied in light clothing, with free access to water. The protocol is shown in Fig. 1. “Arterialized” blood samples were collected from a heated hand by using a 20-gauge intravenous catheter inserted in retrograde fashion in a dorsal hand vein and kept patent by infusion of 0.9% saline. Two additional intravenous cannulae were inserted into the contralateral arm for infusion of insulin, 20% dextrose, and tracers. Subjects received primed infusions of 1,1,2,3,3-^2^H_5_-glycerol (1.6 μmol/kg for 1 min, then 0.11 μmol·kg^−1^·min^−1^) and 6,6-^2^H_2_-glucose (17.6 μmol/kg for 1 min, then 0.22 μmol·kg^−1^·min^−1^), as well as ^13^C_1_-palmitate infused at 0.04 μmol·kg^−1^·min^−1^. Given the short time required to reach steady state with the palmitate tracer, a priming dose was not required and the palmitate infusion was interrupted during from 90–150 min to reduce the volume of 20% albumin infused. Blood samples were taken at baseline, between 60–90 min, and at 210–240 min, the latter during the final 30 min of a low-dose (10 mU·m ^−2^·min^−1^) hyperinsulinemic-euglycemic clamp to investigate suppression of lipolysis and endogenous glucose production. The insulin infusion was primed at 20 mU·m ^−2^·min^−1^ for 10 min, and euglycemia was maintained by a variable-rate infusion of 20% dextrose if necessary. Breath samples were collected immediately before and after an indirect calorimetry hood was applied by use of sealable glass vials (Exetainers, PDZ, Cheshire, UK). Because of a technical problem, the second palmitate infusion was not administered during a single visit for one volunteer.

Each subject emptied his bladder at 0800 and a timed urine collection was performed during the study visit. Aliquots of urine were stored at −20°C and urine nitrogen content determined by the microkjeldahl method (13). Blood was collected in lithium heparin tubes on ice and promptly centrifuged, and plasma was stored at −80°C until analysis.

#### Magnetic resonance spectroscopy.

On another occasion, all subjects underwent proton MRS to quantify intrahepatic triglycerides. Spectra corresponding to water and the methylene [-(CH_2_)_n_-] component of intracellular lipid were obtained and the hepatic fat fraction content calculated as described by Seppala-Lindroos et al. (36) and Szczepaniak et al. (38). Patients were divided into two equal groups based on the median hepatic fat fraction (8.9%): high liver fat (triglyceride content >10.9%) and low liver fat (<6.9%).

#### Preparation of stable isotope tracer infusions.

Stable isotopes were purchased from Cambridge Isotopes Limited (CK gas, Hook, UK). All infusates were passed through a 0.22-μm filter (Millipore, Watford, UK) before infusion. The ^13^C_1_-palmitate infusion was prepared by dissolving its potassium salt in heated water, prior to dissolving in 20% human albumin solution (Zenalb, Scottish National Blood Transfusion Service). 1,1,2,3,3-^2^H_5_-glycerol and 6,6-^2^H_2_-glucose were dissolved in 0.9% saline.

#### Tracer analysis.

To determine isotopic enrichment, plasma FFAs were extracted and derivatized to their methyl esters and analyzed on a ThermoFinnigan *Voyager* gas chromatograph mass spectrometer (GCMS) with an Agilent HP-Innowax column (30 m, 0.320 mm, 0.25 μm) operated in electron impact (EI) ionization mode (70 eV). Source, interface, and injection temperatures were 200, 250, and 260°C, respectively, with selective ion monitoring of molecular ions with *m*/*z* 270, 271, 284, corresponding to [M+0] and [M+1] isotopomers of methyl palmitate and the methyl heptadecanoate internal standard. Glucose and glycerol isotopomers were derivatized to glucose pentacetate and glycerol triacetate, respectively, by use of pyridine:acetic anhydride (1:1 vol/vol) prior to analysis using the same column and GCMS in negative chemical ionization mode. Dilution was required for analysis of glucose, with selective ion monitoring at *m*/*z* of 287 and 289, corresponding to [M+0] and [M+2] fragments of glucose pentacetate. [M+0] and [M+5] isotopomers of glycerol triacetate were measured at *m*/*z* of 217 and 222, respectively. Glycerol concentrations were determined by GCMS using butanetriol as an internal standard. RU38486 levels were measured as previously described (40). Breath ^13^CO_2_ enrichment was measured by isotope ratio mass spectrometry (IRMS). An AP2003 IRMS (Analytical Precision, Northwich, UK) was used to measure ^13^C abundance in each sample with respect to a CO_2_ reference gas that had been calibrated against an international reference.

#### Tracer kinetic calculations.

The rate of appearance (Ra) of palmitate and glycerol was determined by using Steele's equation as applied to steady state, i.e., Ra = tracer infusion rate/TTR, where the TTR is the isotopic enrichment determined by GCMS expressed as the tracer-to-tracee ratio. Isotopic enrichment was deemed to be at steady state if the slope of the TTRs over time, as determined by linear regression, did not significantly differ from zero in each treatment group. Results were expressed per kilogram of fat-free mass (FFM).

The Ra of FFAs was calculated by multiplying the Ra of palmitate by the ratio of plasma FFAs:palmitate (30). Adipose tissue insulin sensitivity was estimated from the percentage suppression from baseline of the Ra of FFAs and glycerol during hyperinsulinemia. Whole body rates of fatty acid reesterification were assessed by using the formula Ra of FFAs − rate of FFA oxidation (determined by indirect calorimetry). Negative calculated values, including percentage suppression, were assigned a value of zero.

The percentage of the infused palmitate tracer oxidized was calculated by using the equation (ECO_2_ * V̇co_2_)/(F * C), where ECO_2_ is the enrichment of expired CO_2 _(corrected for background abundance), V̇co_2_ is the flow rate of expired CO_2_, F is the tracer infusion rate, and C is the bicarbonate correction factor (81%) taking into account nonexcreted carbon. Plasma-derived fatty acid oxidation was calculated by using the equation Ra FFAs * % palmitate oxidized (3).

The Ra of glucose was calculated in basal samples by using Steele's equation modified for the nonsteady state, with a pool fraction of 0.65 and a volume of distribution of 125 ml/kg. Steady-state calculations were used during the final 210- to 240-min sampling period. The rate of endogenous glucose production (EGP) was calculated by subtracting the average glucose infusion rate (GIR) during the final 30 min of the low-dose clamp from the measured Ra of glucose. Hepatic insulin sensitivity was estimated as the percentage suppression from baseline of EGP during low-dose hyperinsulinemia. The hepatic insulin resistance index, an alternative validated measure of hepatic insulin sensitivity for glucose metabolism, was calculated by multiplying the basal Ra glucose by the insulin concentration (28).

M values, or mean GIRs (μmol·kg FFM^−1^·min^−1^) during the final 30 min of the hyperinsulinemic clamp, were corrected for steady-state insulin concentrations (nmol/l) to provide an index of peripheral insulin sensitivity (M/I). The insulin clearance rate (l·m ^−2^·min^−1^) was calculated by dividing the insulin infusion rate (mU·m ^−2^·min^−1^) by the steady-state insulin concentration (mU/l) during the final sampling period.

#### Indirect calorimetry.

A ventilated canopy indirect calorimeter (Europa Gas Exchange Monitor, NutrEn Technology, Cheshire, UK) was used during the three 30-min sampling periods. Rates of fat oxidation were calculated by use of published stoichiometric equations and multiplied by a factor of three to convert to fatty acid oxidation on the basis that 1 mol of triglyceride contains 3 mol of FFAs (14).

#### Other biochemical assays.

ELISA kits were used to determine plasma insulin (DRG Instruments, Marburg, Germany), ACTH (Biomerica), and cortisol (DRG Instruments). Plasma FFAs were measured by a colorimetric assay (Zen Bio, Research Triangle Park, NC) and glucose was measured by the glucose oxidase method.

#### Statistical analysis.

An a priori power calculation showed that a group size of *n* = 8 would be sufficient to detect 30% differences in tracer turnover with 80% power to *P* < 0.05. Thus *n* = 14 per group is generously powered to detect the primary end points of effects of glucocorticoid blockade. The secondary analysis, testing for differences in effects of glucocorticoid blockade in patients with high or low liver fat content, is exploratory. Results are shown as means ± SE. Baseline characteristics of patients with high and low liver fat were compared via independent sample *t*-tests. When comparing a single variable, e.g., fasting glucose, the effect of glucocorticoid blockade in individuals with high and low fatty liver was determined by use of a generalized linear model for repeated measures. Similarly, when comparing measurements at repeated time points, e.g., Ra of FFAs before and during hyperinsulinemia, a generalized linear model was used to determine the effects of glucocorticoid blockade and insulin (both within-subject variables), and liver fat (a between-subjects variable), and any interactions determined by multivariate testing and tests of within-subject effects by use of SPSS (IBM).

## RESULTS

### 

#### Baseline characteristics.

Table 1 shows baseline patient characteristics, including intrahepatic triglyceride content of the high and low liver fat subgroups. There were trends for higher body weight, fat mass, and waist circumference in individuals with high liver fat, but age, time since diagnosis of diabetes, and biochemical parameters were similar. There was no significant difference in metformin dose between subgroups (data not shown).

**Table 1.** Baseline characteristics and fasting plasma biochemical indexes of study participants with high vs. low liver fat

	All Patients	High Liver Fat	Low Liver Fat
Number of patients	14	7	7
Liver fat, %	13.7 ± 3.2	23.0 ± 3.9^a^	4.4 ± 0.8
Age, yr	58.6 ± 1.7	59.3 ± 2.6	58.0 ± 2.2
Weight, kg	97.6 ± 3.4	101.8 ± 5.1	93.0 ± 4.4
Body mass index, BMI, kg/m^2^	31.9 ± 1.2	34.2 ± 2	29.7 ± 1.1
Waist circumference, cm	110.4 ± 2.5	114.7 ± 3.6	106.2 ± 3.0
Fat mass, kg	30.0 ± 2.1	33.6 ± 3.1	26.5 ± 2.2
Fat-free mass, kg	67.7 ± 1.6	68.2 ± 2.4	67 ± 2.4
Time since diagnosis of diabetes, yr	5.3 ± 1.0	4.8 ± 1.4	5.8 ± 1.6
HbA1c, % [mmol/mol]	6.8 ± 0.2 (51 ± 2.2)	7.0 ± 0.2 (53 ± 2.2)	6.5 ± 0.4 (48 ± 4.4)
ALT, IU/l	40.9 ± 2.7	45.1 ± 3.4	36.6 ± 3.8
ALP, IU/l	74.5 ± 4.7	79.4 ± 7.2	69.6 ± 6.0
GGT, IU/l	21.9 ± 6.1	18.3 ± 8.9	26.2 ± 8.8
Total cholesterol, mmol/l	3.9 ± 0.1	4.0 ± 0.1	3.8 ± 0.17
Triglycerides, mmol/l	1.9 ± 0.3	2.2 ± 0.5	1.6 ± 0.29
HDL cholesterol, mmol/l	1.0 ± 0.5	1.0 ± 0.1	1.0 ± 0.03
LDL cholesterol, mmol/l	2.0 ± 0.2	1.8 ± 0.3	2.1 ± 0.2
Total-to-HDL cholesterol ratio	3.9 ± 0.2	4.0 ± 0.3	3.7 ± 0.2

Results are mean±SE. Groups were compared using independent sample *t*-tests (equal variances not assumed):

a *P* < 0.01. ALT, alanine aminotransferase; ALP, alkaline phosphatase; GGT, gamma glutamyltransferase; HDL, high-density lipoprotein; LDL, low-density lipoprotein.

#### ACTH and cortisol levels.

**Fig. 2.**
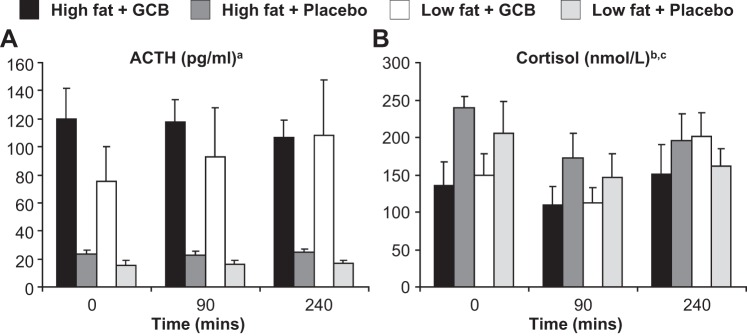
Effects of glucocorticoid blockade (GCB) on plasma ACTH and cortisol. The effect of GCB on plasma ACTH (*A*) and cortisol (*B*) levels in patients with Type 2 diabetes and high or low liver fat (*n* = 7/group). Results are means ± SE. Effects of glucocorticoid blockade and/or liver fat were analyzed by use of a generalized linear model for repeated measures with liver fat as a between-subjects factor. ^a^GCB increased ACTH in all subjects (*P* < 0.0001) with no effect of liver fat or insulin/time. ^b^Cortisol levels changed over time (*P* < 0.005; *P* < 0.01 for 0 vs. 90 min and *P* < 0.05 for 90 vs. 240 min on least significant difference post hoc testing) and tended to decrease with glucocorticoid blockade (*P* = 0.075 for glucocorticoid blockade). ^c^*P* < 0.05 for an interaction of glucocorticoid blockade with time/insulin.

Glucocorticoid blockade increased plasma ACTH (Fig. 2*A*), whereas metyrapone prevented any compensatory increase in plasma cortisol (Fig. 2*B*) and produced a modest but nonsignificant overall reduction in cortisol levels (*P* = 0.075). ACTH levels were stable throughout, whereas, after an initial fall, cortisol levels increased toward the end of each study day, moreso in those receiving glucocorticoid blockade (*P* < 0.05 for an interaction between glucocorticoid blockade and time). This suggests the effect of metyrapone may have declined by the end of the protocol, whilst importantly, the GR blocking effect of RU38486 was maintained throughout. ACTH and cortisol levels were similar in patients with high and low liver fat after both placebo and glucocorticoid blockade (Fig. 2*B*). RU38486 concentrations were similar following glucocorticoid blockade (2.8 ± 0.5 vs. 2.8 ± 0.5 μmol/l in high vs. low liver fat groups), and undetectable following placebo.

#### Glucose metabolism.

**Fig. 3.**
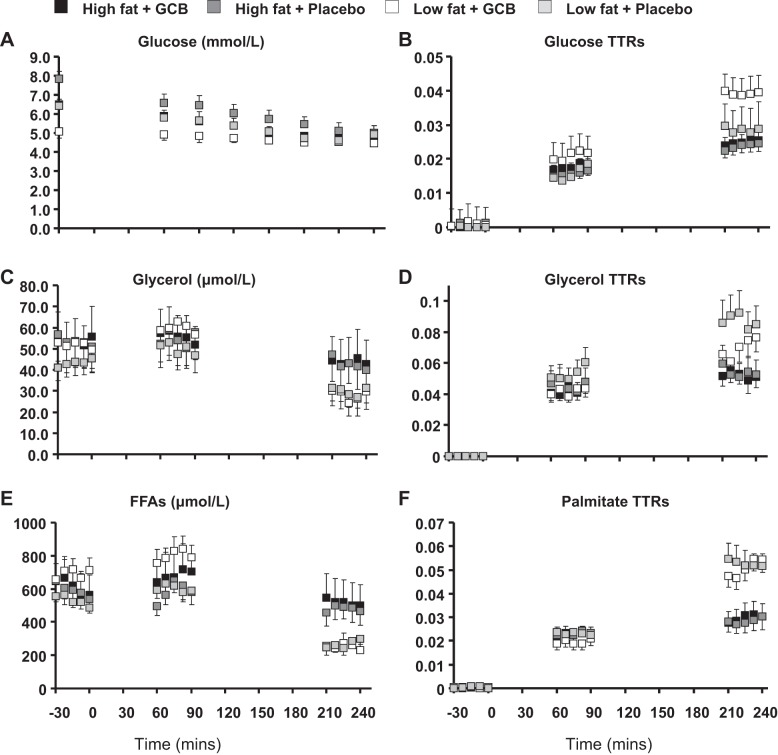
Metabolic effects of glucocorticoid blockade. Effects of glucocorticoid blockade in high and low liver fat subgroups on plasma glucose concentration (*A*), D2-glucose enrichment (*B*), plasma glycerol concentration (*C*), D5-glycerol enrichment (*D*), plasma free fatty acid (FFA) concentration (*E*), and ^13^C-palmitate enrichment (*F*). Results are means ± SE. *N* = ∼7/group. Statistical analyses of mean concentrations and kinetic calculations using mean tracer-to-tracee ratios (TTRs) are shown in Tables 2 and 3.

Plasma glucose profiles, and glucose TTRs are shown in Fig. 3, *A* and *B*, and results of kinetic calculations in Tables 2 and 3. During the low-dose insulin infusion there was a modest but significant rise in insulin levels, which suppressed EGP.

**Table 2.** Effects of glucocorticoid blockade on glucose metabolism in all subjects

	All Patients (*n* = 14)		
	GCB	Placebo	*P* Value Effect of GCB
Plasma glucose, mmol/l^a^			
Fasting	5.8 ± 0.2	7.1 ± 0.3	<0.0001
Hyperinsulinemia	4.6 ± 0.1	4.8 ± 0.2	0.30
Plasma insulin, pmol/l^e^			
Fasting	78.6 ± 16.8	102.6 ± 17.7	<0.05^b^
Prolonged fasting	59.3 ± 10.2	74.2 ± 11.3	<0.05^c^
Hyperinsulinemia	100.1 ± 8.8	114.2 ± 11.0	
Insulin clearance, l·m^−2^·min^−1^	0.78 ± 0.08	0.70 ± 0.07	0.10
M value, μmol·kg FFM·min^−1^	4.7 ± 1.0	3.3 ± 1.2	0.28
M/I index, μmol·kg FFM^−1^·min^−1^·nmol^−1^·l^−1^	59.4 ± 15.0	43.8 ± 21.0	0.46
EGP, μmol·kg FFM^−1^·min^−1^^d^			
Prolonged fasting	16.5 ± 1.8	19.0 ± 2.3	0.05
Hyperinsulinemia	10.7 ± 1.4	12.4 ± 1.1	
% Suppression	34.9 ± 6.4	29.3 ± 6.2	0.41
Hepatic IR index, pmol·l^−1^·mmol^−1^·kg FFM^−1^·min^−1^	1.0 ± 0.2	1.4 ± 0.2	<0.001
Glucose oxidation by indirect calorimetry, μmol·kg FFM^−1^·min^−1^^e^			
Fasting	13.7 ± 1.2	13.5 ± 1.2	0.60
Prolonged fasting	10.5 ± 0.9	11.3 ± 0.6	
Hyperinsulinemia	9.4 ± 1.1	10.5 ± 1.4	

Results are means ± SE. Fasting refers to mean of measurements from −30 to 0 min, Prolonged fasting to +60 to 90 min, and Hyperinsulinemia to +210 to 240 min in the protocol. EGP, endogenous glucose production; FFM, fat-free mass; GCB, glucocorticoid blockade; M value; mean glucose infusion rate during 210–240 min; IR, insulin resistance. To determine any overall effect of glucocorticoid blockade, at all time points measured, in patients with and without increased liver fat, data were analyzed using a generalized linear model for repeated measures, with liver fat as a between-subjects variable. *P* values are derived from the generalized linear models for the effects of GCB and/or of liver fat. Footnotes denote any additional significant interactions of glucocorticoid blockade, liver fat, and insulin infusion (or time) as determined by multivariate testing and tests of within-subject effects.

a Fasting and hyperinsulinemic plasma glucose concentrations were analyzed separately.

b *P* value for analysis of fasting insulin concentrations alone.

c *P* < 0.05 for an interaction between GCB or liver fat and time/insulin.

d *P* < 0.01 for an effect of insulin/time.

e *P* < 0.001 for an effect of insulin/time.

**Table 3.** Effects of glucocorticoid blockade on glucose metabolism in subjects classified by liver fat content

	High Liver Fat (*n* = 7)		Low Liver Fat (*n* = 7)		*P* Values	
	GCB	Placebo	GCB	Placebo	Effect of GCB	Effect of liver fat
Plasma glucose, mmol/l^a^						
Fasting	6.5 ± 0.1	7.8 ± 0.4	5.1 ± 0.4	6.4 ± 0.4	<0.0001	<0.01
Hyperinsulinemia	4.8 ± 0.1	5.1 ± 0.4	4.5 ± 0.1	4.6 ± 0.2	0.30	0.27
Plasma insulin, pmol/l^e^						
Fasting	121 ± 33	149 ± 35	36 ± 6	56 ± 7	<0.05^b^	<0.05^b^
Prolonged fasting	87 ± 20	106 ± 22	31 ± 4	42 ± 5	<0.05^c^	<0.05^c^
Hyperinsulinemia	113 ± 14	135 ± 19	87 ± 11	93 ± 11		
Insulin clearance, l·m^−2^·min	0.67 ± 0.11	0.58 ± 0.1	0.89 ± 0.12	0.82 ± 0.10	0.10	0.12
M value, μmol·kg FFM^−1^·min^−1^	2.3 ± 1.3	0.7 ± 0.4	7.1 ± 1.4	5.9 ± 2.3	0.28	<0.05
M/I index, μmol·kg FFM^−1^·min^−1^·nmol^−1^·l^−1^	25.2 ± 16.2	7.4 ± 6.0	93.7 ± 25.3	80.3 ± 41.5	0.46	<0.05
EGP, μmol·kg FFM^−1^·min^−1^^d^						
Prolonged fasting	17.2 ± 2.1	19.3 ± 2.4	15.7 ± 2.9	18.7 ± 3.8	0.05	0.35
Hyperinsulinemia	12.9 ± 1.3	14.0 ± 1.4	8.5 ± 2.5	10.8 ± 1.6		
% Suppression	23.0 ± 4.7	25.5 ± 4.4	45.8 ± 12.1	31.8 ± 11.7	0.41	0.20
Hepatic IR index, pmol·l^−1^·mmol^−1^·kg FFM^−1^·min^−1^	1.5 ± 0.4	2.0 ± 0.4	0.5 ± 0.1	0.7 ± 0.1	<0.001	<0.05
Glucose oxidation by indirect calorimetry, μmol·kg FFM^−1^·min^−1^^e^						
Fasting	15.0 ± 2.1	13.8 ± 1.3	12.4 ± 1.4	13.2 ± 2.0	0.60	0.71^c^
Prolonged fasting	11.3 ± 1.7	11.0 ± 1.0	9.7 ± 0.5	11.7 ± 0.7		
Hyperinsulinemia	8.8 ± 0.9	7.7 ± 1.7	10.1 ± 2.0	13.3 ± 2.1		

Results are means ± SE. Fasting refers to mean of measurements from −30 to 0 min, Prolonged fasting to +60 to 90 min, and Hyperinsulinemia to +210 to 240 min in the protocol. To determine any overall effect of glucocorticoid blockade, at all time points measured, in patients with and without increased liver fat, data were analyzed using a generalized linear model for repeated measures, with liver fat as a between-subjects variable. *P* values are derived from the generalized linear models for the effects of GCB and/or of liver fat. Footnotes denote any additional significant interactions of glucocorticoid blockade, liver fat, and insulin infusion (or time) as determined by multivariate testing and tests of within-subject effects.

a Fasting and hyperinsulinemic plasma glucose concentrations were analyzed separately.

b *P* value for analysis of fasting insulin concentrations alone.

c *P* < 0.05 for an interaction between GCB or liver fat and time/insulin.

d *P* < 0.01 for an effect of insulin/time.

e *P* < 0.001 for an effect of insulin/time.

Glucocorticoid blockade lowered fasting insulin and glucose concentrations, as well as insulin levels throughout the protocol, although there was no significant increase in insulin clearance, and plasma glucose was successfully maintained at similar levels during the final 30 min of the low-dose hyperinsulinemic clamp. Glucocorticoid blockade decreased EGP (*P* = 0.05), reducing the corresponding hepatic insulin resistance index, but had no influence on glucose oxidation.

None of the effects of GC blockade was measurably different between high and low liver fat subgroups, i.e., there were no interactions between glucocorticoid blockade and liver fat in the general linear model analyses. However, individuals with Type 2 diabetes and high liver fat had higher fasting glucose and insulin levels, lower GIRs, and a corresponding higher M/I index and sustained higher insulin levels throughout the protocol than those with low liver fat. Glucose levels during the final 30 min of the clamp and insulin clearance did not differ between liver fat subgroups. As a result, although EGP and its suppression by insulin did not significantly differ between subgroups, the hepatic insulin resistance index was higher in the high liver fat subgroup. High liver fat had no influence on basal rates of glucose oxidation but was associated with a failure to maintain rates of glucose oxidation during hyperinsulinemia (Table 3).

#### Fatty acid metabolism.

Plasma glycerol and FFA concentrations and TTRs are displayed in Fig. 3, *C*–*F*, and statistical analyses of the mean values in steady state with and without insulin infusion are shown in Tables 4 and 5. Low-dose insulin infusion suppressed glycerol and FFA concentrations, the Ra of glycerol and Ra FFAs in plasma, and rates of FFA oxidation and reesterification.

**Table 4.** Effects of glucocorticoid blockade on fatty acid metabolism in all subjects

	All Patients (*n* = 14)		
	GCB	Placebo	*P* Value Effect of GCB
Plasma glycerol, μmol/l^a^			
Fasting	53 ± 8	48 ± 7	0.21
Prolonged fasting	58 ± 7	52 ± 7	
Hyperinsulinemia	36 ± 7	36 ± 6	
Plasma FFAs, mmol/l^a^			
Fasting	653 ± 64	561 ± 35	0.06^c^
Prolonged fasting	740 ± 80	592 ± 40	
Hyperinsulinemia	386 ± 68	374 ± 45	
Ra Glycerol, μmol·kg FFM^−1^·min^−1^^a^			
Prolonged fasting	4.7 ± 0.4	4.2 ± 0.6	0.49^c^
Hyperinsulinemia	3.3 ± 0.3	3.3 ± 0.3	
% Suppression	29.9 ± 2.7	21.6 ± 3.7	0.05
Ra FFAs, μmol·kg FFM^−1^·min^−1^^b^			
Prolonged fasting	9.6 ± 4.2	7.6 ± 1.0	0.27
Hyperinsulinemia	4.5 ± 0.9^§^	4.6 ± 0.6	
% Suppression	49.1 ± 7.9^§^	35.2 ± 7.0	0.06
FFA oxidation by indirect calorimetry, μmol·kg FFM^−1^·min^−1^^b^			
Fasting	3.3 ± 0.3	3.2 ± 0.2	0.35
Prolonged fasting	4.1 ± 0.2	3.8 ± 0.3	
Hyperinsulinemia	4.3 ± 0.3	3.9 ± 0.4	
% Suppression	10.7 ± 4.8	13.6 ± 5.2	0.65
Plasma FFA oxidation^§§^, μmol·kg FFM^−1^·min^−1^^b^			
60 min	0.41 ± 0.06	0.33 ± 0.06	0.77^c^
90 min	0.54 ± 0.06	0.48 ± 0.07	
210 min	0.41 ± 0.05	0.41 ± 0.06	
240 min	0.51 ± 0.06	0.50 ± 0.07	
% Suppression, 90 vs. 210 min	26.7 ± 7.6	20.5 ± 7.2	0.41
FFA reesterification, μmol·kg FFM^−1^·min^−1^^b^			
Prolonged fasting	5.5 ± 1.2	3.8 ± 1.0	0.48
Hyperinsulinemia	1.2 ± 0.8	1.2 ± 0.8	
% Suppression	79 ± 11	70 ± 11	0.63

Data are means ± SE. *N* = 7/group except where ^§^*n* = 6, ^§§^*n* = 4–6. Fasting refers to measurements at −30 to 0 min, Prolonged fasting to +60 to 90 min, and Hyperinsulinemia to +210 to 240 min in the protocol; % Suppression describes comparison of hyperinsulinemic with basal conditions. FFM, fat-free mass; GCB, glucocorticoid blockade; NS, not significant; Ra, rate of appearance. To determine any overall effect of glucocorticoid blockade, at all time points measured, in patients with and without increased liver fat, data were analyzed using a generalized linear model for repeated measures, with liver fat as a between-subjects variable. *P* values are derived from the generalized linear models for the effects of GCB and/or of liver fat. Footnotes denote any additional significant interactions of glucocorticoid blockade, liver fat and insulin infusion (or time) as determined by multivariate testing and tests of within-subject effects.

a *P* < 0.0001 for an effect of insulin/time.

b *P* < 0.01 for an effect of insulin/time.

c *P* < 0.05 for an interaction of GCB or liver fat with insulin/time.

d *P* < 0.01 for an interaction of GCB or liver fat with insulin/time.

e *P* < 0.001 for an interaction of GCB or liver fat with insulin/time.

**Table 5.** Effects of glucocorticoid blockade on fatty acid metabolism in subjects classified by liver fat content

	High Liver Fat (*n* = 7)		Low Liver Fat (*n* = 7)		*P* Values	
	GCB	Placebo	GCB	Placebo	Effect of GCB	Effect of liver fat
Plasma glycerol, μmol/l^a^						
Fasting	54 ± 11	54 ± 12	52 ± 12	43 ± 6	0.21	0.58
Prolonged fasting	56 ± 10	54 ± 11	60 ± 10	50 ± 8		
Hyperinsulinemia	44 ± 12	43 ± 10	28 ± 8	30 ± 6		
Plasma FFAs, mmol/l^a^						
Fasting	612 ± 117	593 ± 60	694 ± 67	529 ± 42	0.06^c^	0.60^e^
Prolonged fasting	679 ± 148	582 ± 72	801 ± 75	603 ± 47		
Hyperinsulinemia	518 ± 135	480 ± 83	253 ± 33	268 ± 45		
Ra glycerol, μmol·kg FFM^−1^·min^−1^^a^						
Prolonged fasting	4.6 ± 0.5	4.8 ± 1.0	4.9 ± 0.7	3.6 ± 0.6	0.49^c^	0.30^c^
Hyperinsulinemia	3.8 ± 0.5	4.3 ± 1.0	2.7 ± 0.2	2.4 ± 0.4		
% Suppression	17.2 ± 4.2	10.6 ± 5.1	42.6 ± 3.3	32.5 ± 5.4	0.05	<0.001
Ra FFAs, μmol·kg FFM^−1^·min^−1^^b^						
Prolonged fasting	9.3 ± 2.3	7.0 ± 0.6	9.9 ± 1.2	8.2 ± 1.9	0.27	0.69^d^
Hyperinsulinemia	6.3 ± 1.5^§^	6.1 ± 0.7	3.0 ± 0.5	3.1 ± 0.6		
% Suppression	27.2 ± 9.3^§^	15.9 ± 6.0	66.0 ± 7.7	54.4 ± 8.2	0.06	<0.001
FFA oxidation by indirect calorimetry, μmol·kg FFM^−1^·min^−1b^						
Fasting	3.2 ± 0.4	3.5 ± 0.5	3.2 ± 0.4	3.0 ± 0.4	0.35	0.14^c^
Prolonged fasting	4.2 ± 0.3	4.2 ± 0.4	4.1 ± 0.2	3.4 ± 0.2		
Hyperinsulinemia	4.7 ± 0.4	5.0 ± 0.7	3.9 ± 0.6	2.8 ± 0.4		
% Suppression	4.1 ± 4.1	6.5 ± 3.7	17.4 ± 8.3	20.6 ± 9.0	0.65	0.08
Plasma FFA oxidation^§§^, μmol·kg FFM^−1^·min^−1^^b^						
60 min	0.37 ± 0.08	0.30 ± 0.07	0.45 ± 0.08	0.37 ± 0.09	0.77^c^	0.70^d^
90 min	0.43 ± 0.07	0.40 ± 0.06	0.65 ± 0.09	0.58 ± 0.12		
210 min	0.48 ± 0.07	0.49 ± 0.08	0.35 ± 0.05	0.31 ± 0.07		
240 min	0.60 ± 0.09	0.57 ± 0.09	0.41 ± 0.08	0.40 ± 0.11		
% Suppression, 90 vs. 210 min	4.5 ± 2.7	3.8 ± 4.1	32.8 ± 12.5	31.1 ± 12.8	0.41	<0.001
FFA reesterification, μmol·kg FFM^−1^·min^−1b^						
Prolonged fasting	5.2 ± 2.2	2.7 ± 0.8	5.8 ± 1.1	4.8 ± 1.8	0.48	0.90^c^
Hyperinsulinemia	2.5 ± 1.6	2.0 ± 1.0^§^	0.1 ± 0.1	0.4 ± 0.3		
% Suppression	57.1 ± 18.7	56.1 ± 18.5^§^	98.3 ± 1.6	84.0 ± 11.6	0.63	0.05

Data are means ± SE. *N* = 7/group except where ^§^*n* = 6, ^§§^*n* = 4–6. Fasting refers to measurements at −30 to 0 min, Prolonged fasting to +60 to 90 min, and Hyperinsulinemia to +210 to 240 min in the protocol; % Suppression describes comparison of hyperinsulinemic with basal conditions. To determine any overall effect of glucocorticoid blockade, at all time points measured, in patients with and without increased liver fat, data were analyzed using a generalized linear model for repeated measures, with liver fat as a between-subjects variable. *P* values are derived from the generalized linear models for the effects of GCB and/or of liver fat. Footnotes denote any additional significant interactions of glucocorticoid blockade, liver fat and insulin infusion (or time) as determined by multivariate testing and tests of within-subject effects.

a *P* < 0.0001 for an effect of insulin/time.

b *P* < 0.01 for an effect of insulin/time.

c *P* < 0.05 for an interaction of GCB or liver fat with insulin/time.

d *P* < 0.01 for an interaction of GCB or liver fat with insulin/time.

e *P* < 0.001 for an interaction of GCB or liver fat with insulin/time.

Glucocorticoid blockade had modest effects on fatty acid metabolism, with little effect on baseline indexes, but potentiated insulin-mediated suppression of FFA concentrations and Ra glycerol, also tending to increase suppression of Ra FFAs. Glucocorticoid blockade had no effects on FFA oxidation or reesterification. There were no interactions between glucocorticoid blockade and liver fat on measures of glycerol or FFA turnover to suggest any differential effect of glucocorticoid blockade in patients with high liver fat.

The high liver fat subgroup showed striking differences in fatty acid metabolism compared with patients with low liver fat, most evident in their responsiveness to low-dose insulin infusion, with impaired suppression of FFA and glycerol concentrations and rates of appearance, as well as suppression of fatty acid oxidation and reesterification.

## DISCUSSION

These detailed metabolic studies show that blocking cortisol action in patients with Type 2 diabetes has significant metabolic effects, leading to insulin sensitization in both adipose tissue and the liver, with associated reductions in fasting plasma glucose concentrations and hyperinsulinemia. These effects appear to be independent of baseline liver fat content, since similar effects of glucocorticoid blockade were observed in patients with high or low liver fat, albeit this inference is drawn from a secondary subgroup analysis. In addition, the measurements made at baseline and during the placebo phase allowed us to detail the metabolic differences between patients with and without high liver fat content; this is the first such report among patients with Type 2 diabetes. The high liver fat group exhibited similar manifestations of more severe insulin resistance for both fatty acid and glucose metabolism than patients with low liver fat, as previously reported in nondiabetic patients with NAFLD (5, 9, 11).

Although there are extensive previous studies describing the effects of elevated glucocorticoids on metabolism, very few have addressed the consequences of reducing cortisol action, particularly in the context of diabetes (reviewed in Refs. 1, 25). In studies in healthy subjects using limited assessments of metabolic flux, glucocorticoid receptor antagonism with RU38486 has been shown to achieve substantial drug levels in adipose tissue (17) and to lower serum triglycerides (31, 40) and reduce EGP (15). We show that glucocorticoid blockade was technically successful in elevating ACTH and preventing significant rebound hypercortisolemia (Fig. 2), resulting in lower fasting glucose and insulin levels and improved insulin sensitivity at multiple sites.

The influence of glucocorticoids on hepatic gluconeogenesis is well recognized, although the mechanisms are incompletely defined, with both indirect effects of FFAs and direct effects of cortisol potentially promoting gluconeogenesis (reviewed in Refs. 1, 25). Here, the lack of any reduction in basal FFA turnover associated with the fall in EGP suggests direct effects of glucocorticoids on the liver prevail, in keeping with recent evidence using 11β-HSD1 inhibitors in dogs (10).

The mechanism for the improvement in adipose tissue insulin sensitivity following glucocorticoid blockade is complex. In vitro data suggest that glucocorticoids have a permissive effect to increase turnover between FFAs and triglyceride in adipocytes by inducing both lipolysis and reesterification (25, 33). In vivo studies have suggested that the net influence of cortisol on lipolysis is highly dependent on prevailing insulin concentrations: cortisol has a more pronounced effect to induce lipolysis when insulin levels are low, e.g., during a pancreatic clamp or overnight, whereas rates of lipolysis may be reduced by cortisol when there is compensatory hyperinsulinemia, e.g., in Cushing's syndrome. These observations are based on elevated cortisol levels, however, and cannot necessarily be extrapolated when reducing physiological cortisol action. We found that glucocorticoid blockade increased insulin-mediated suppression of glycerol release, with a similar trend for FFA turnover, despite somewhat lower plasma insulin concentrations, but we did not find effects on reesterification of FFA to triglycerides or in the balance of oxidation of FFAs or glucose.

Previous studies employing tracers in patients without diabetes have shown a number of metabolic abnormalities associated with liver fat, including increased FFA and glycerol turnover, impaired suppression of lipolysis by insulin, increased hepatic triglyceride export, de novo lipogenesis, and hepatic insulin resistance (5, 9, 11). Similar metabolic abnormalities characterize patients with Type 2 diabetes (2, 21), although our results show clear differences among those with Type 2 diabetes according to whether they have high or low liver fat. These findings suggest that propensity to liver fat accumulation in Type 2 diabetes is associated with hyperinsulinemia and with more marked adipose, hepatic, and peripheral insulin resistance. Patients with Type 2 diabetes and high liver fat also had impaired metabolic flexibility, with a reduced ability to switch from fat to glucose oxidation during hyperinsulinemia.

The lack of any differential effect of glucocorticoid blockade in the subgroup with high liver fat was unexpected, although this inference is drawn from a subgroup analysis with limited statistical power to detect subtle differences in response between groups. We defined high liver fat on the basis of the median observed hepatic fat fraction in our participants (∼7%) rather than the published criteria of 6.1% (∼5.5% corrected intrahepatic triglyceride content per 100 g wet liver tissue) derived from nondiabetic individuals with no risk factors for NAFLD (39) or the inclusion criteria (>5.56%) for a recently published study of 11β-HSD1 inhibition in NAFLD (37). Since liver fat is continuously distributed it is arguably an oversimplification to regard any cutoff value as definitive, and our definition is consistent with the median liver fat content reported among patients with Type 2 diabetes in Edinburgh, where our participants were recruited (42). By comparing subgroups with a high vs. arguably a low-moderate liver fat content it is possible we may have underestimated differences in the high liver fat subgroup. MRS scans were performed at the time of screening, on average 51 days before the tracer studies, but the intrasubject coefficient of variation of liver fat measurements has previously been estimated to be low (8.5%) (39) and there was no change in body weight between study visits. There was no effect of liver fat on ACTH or RU38486 levels to suggest altered efficacy of the drug regimen. At face value, these findings suggest that whereas tissue-specific changes in glucocorticoid metabolism may be important modulators of liver fat, they may not be the primary driver of increased liver fat in Type 2 diabetes. Nevertheless, a number of metabolic parameters were improved, and the lowering of plasma insulin by glucocorticoid blockade may, of itself, be advantageous since in NAFLD, despite resistance to the effect of insulin to suppress gluconeogenesis, sensitivity to promotion of de novo lipogenesis is retained (24); glucocorticoid blockade may therefore also reduce hepatic lipogenesis, although this was not tested in our acute intervention study.

As with all detailed metabolic studies there are technical limitations to consider. The discrepancy between the hepatic insulin resistance index and the percentage suppression of EGP may have been due to omission of D2-glucose from the 20% dextrose infusion. The plasma-derived FFA oxidation results need to be interpreted with caution because we did not prime the bicarbonate pool, although the results closely mirror the indirect calorimetry data. The high liver fat subgroup also tended to be more obese; we expressed our tracer data, however, per kilogram of fat-free mass, which is thought most closely to represent the flux of FFAs toward lean tissues, including the liver (22). All participants were receiving metformin, which might interact with glucocorticoid blockade but suggests pragmatically that therapies to lower cortisol may be of benefit in patients currently on optimized oral antidiabetic therapy.

In this acute intervention study we demonstrated that reducing glucocorticoid action appears to improve insulin sensitivity at a number of sites in patients with Type 2 diabetes. These actions may contribute to improvements in a range of metabolic variables following longer term reductions in glucocorticoid action resulting from inhibition of 11β-HSD1 (12, 35, 37). Our results suggest glucocorticoid blockade is equally efficacious in individuals with Type 2 diabetes and increased liver fat, despite the multiple sites of greater insulin resistance found in these individuals.

## GRANTS

This work was kindly supported by grants from the British Heart Foundation (B. R. Walker, R. Andrew) and Wellcome Trust (D. P. Macfarlane). The indirect calorimeter was purchased with an MRC JREI grant awarded to Prof. Tom Preston.

## DISCLOSURES

B. R. Walker is an inventor on relevant patents owned by the University of Edinburgh and has consulted for a number of pharmaceutical companies developing new therapies targeted at glucocorticoid action.

## AUTHOR CONTRIBUTIONS

D.P.M., P.J.R., M.E.B., I.M., R.A., and B.R.W. conception and design of research; D.P.M. and T.P. performed experiments; D.P.M., T.P., C.D.G., R.A., and B.R.W. analyzed data; D.P.M., J.P.I., R.A., and B.R.W. interpreted results of experiments; D.P.M. and B.R.W. prepared figures; D.P.M. drafted manuscript; D.P.M., P.J.R., R.A., and B.R.W. edited and revised manuscript; D.P.M., P.J.R., T.P., C.D.G., M.E.B., I.M., J.P.I., R.A., and B.R.W. approved final version of manuscript.
